# Associates of an Elevated Natriuretic Peptide Level in Stable Heart Failure Patients: Implications for Targeted Management

**DOI:** 10.1155/2013/562763

**Published:** 2013-12-25

**Authors:** Aftab Jan, Ian Dawkins, Niamh Murphy, Patrick Collier, John Baugh, Mark Ledwidge, Kenneth McDonald, Chris J. Watson

**Affiliations:** ^1^Chronic Cardiovascular Disease Management Unit, St. Vincent's Healthcare Group/St. Michael's Hospital, Dublin, Ireland; ^2^School of Medicine & Medical Science, UCD Conway Institute, University College Dublin, Dublin 4, Ireland

## Abstract

*Background*. Persistently elevated natriuretic peptide (NP) levels in heart failure (HF) patients are associated with impaired prognosis. Recent work suggests that NP-guided therapy can improve outcome, but the mechanisms behind an elevated BNP remain unclear. Among the potential stimuli for NP in clinically stable patients are persistent occult fluid overload, wall stress, inflammation, fibrosis, and ischemia. The purpose of this study was to identify associates of B-type natriuretic peptide (BNP) in a stable HF population. *Methods*. In a prospective observational study of 179 stable HF patients, the association between BNP and markers of collagen metabolism, inflammation, and Doppler-echocardiographic parameters including left ventricular ejection fraction (LVEF), left atrial volume index (LAVI), and *E/e* prime (*E/e*′) was measured. *Results*. Univariable associates of elevated BNP were age, LVEF, LAVI, *E/e*′, creatinine, and markers of collagen turnover. In a multiple linear regression model, age, creatinine, and LVEF remained significant associates of BNP. *E/e*′ and markers of collagen turnover had a persistent impact on BNP independent of these covariates. *Conclusion*. Multiple variables are associated with persistently elevated BNP levels in stable HF patients. Clarification of the relative importance of NP stimuli may help refine NP-guided therapy, potentially improving outcome for this at-risk population.

## 1. Introduction

Persistently elevated natriuretic peptide (NP) levels in stable heart failure (HF) patients are associated with an increased incidence of cardiovascular events [1–4]. Several datasets have demonstrated that elevated values of this biomarker are associated with increased mortality and more frequent HF-related morbidity, in particular hospitalization [5]. The strength of this association has been so convincing that several studies have been reported on the merit of using NP levels in addition to clinical assessment to guide therapy of patients with stable HF [6–9]. The predominant outcome from these studies to date has been encouraging, with an impact on morbidity and longer-term mortality compared to usual care, albeit confined to those aged less than 75 years.

Notwithstanding these encouraging results, many patients in the active arm of these studies fail to lower their NP levels to less than target value [6, 9] and questions still persist in regard to how best to manage persistently elevated NP levels. Lacking at present is a more complete understanding of what drives the elevated level of the biomarker in a clinically stable patient. Based on the clinical and experimental literature, it is possible that varied stimuli may be involved. Whereas BNP is classically known to respond to pressure and volume overload, it has been shown that there are other associates of BNP including increased wall stress, inflammation, fibrosis, atrial fibrillation, and ischemia [10–14]. Furthermore, the absence of clinical signs of fluid overload in stable patients with elevated NP levels does not exclude occult congestion [15]. In addition, elevated NP levels may be partly explained by renal dysfunction [16], by production of less active alternative forms of the NP protein [17, 18], or even from congenital structural abnormalities [19]. Therefore, it may be preferable and more effective to adopt management strategies that are more specific to the underlying stimuli of the elevated NP level.

The purpose of this study is to investigate factors associated with an elevated NP level in a stable HF population through analysis of markers of structural abnormality of the heart, fluid overload, inflammation, and fibrosis.

## 2. Materials and Methods

This report is a prospective observational study of stable HF patients who participated in a HF disease management programme (DMP) and attended the HF unit for annual review. All subjects gave written informed consent to participate in the study. The Ethics Committee at St. Vincent's University Hospital approved the study protocol and has therefore been performed in accordance with the ethical standards laid down in the 1964 Declaration of Helsinki and its later amendments.

Annual review of all patients is a standard feature of the DMP to ensure clinical stability and review treatment strategies. For the purpose of this study, we included all patients attending for an annual review who were clinically stable for 1 month before enrolment (as defined by freedom from an episode of clinical deterioration or change in HF-related medication). BNP and clinical parameters at the time of annual review visit are included on the DMP database which also includes patient demographics, past medical history, and details of prior visits to the DMP.

All patients had appropriate clinical and laboratory evaluation to identify exclusion criteria and suitability for the study. Patients with conditions known to alter collagen turnover, including chronic liver disease, connective tissue disorders, metabolic bone diseases, and malignancy and those who underwent recent trauma or surgery (<6 months) were excluded from this protocol. Patients with active infection were also excluded.

### 2.1. BNP Analysis

BNP measurements were performed on nonfasting venous blood samples which were drawn into vacutainers containing EDTA. All samples were analysed using the BioSite point of care meters (Triage).

### 2.2. Biochemical Measurements of Indices of Collagen Metabolism and Inflammation

Peripheral venous blood samples were drawn during clinical assessment and immediately underwent serum isolation. Each sample was centrifuged at 1200 g for 10 minutes at 4°C. The serum was then separated into aliquots and stored at –80°C until required for analysis. Aminoterminal propeptide of procollagen type I (PINP) and type III (PIIINP) and carboxyterminal telopeptide of collagen type I (CITP) were measured by radioimmunoassay with commercial antiserum kits (Orion Diagnostica). The intra-assay variations for determining PINP, PIIINP, and CITP were 7%, <5%, and <8%, respectively. The sensitivities (lower detection limit) of the assays for PINP, PIIINP, and CITP were 13 *μ*g/L, 1.9 *μ*g/L, and 0.5 *μ*g/L, respectively. Carboxyterminal propeptide of procollagen type I (PICP) was measured with a specific enzyme-linked immunosorbent assay based on the manufacturer's method (Takara Biochemicals). The sensitivity for PICP was 2 ng/mL.

Serum matrix metalloproteinase (MMP) types 2 and 9 and tissue inhibitors of matrix metalloproteinase (TIMP) were analyzed using commercially available 2-site sandwich enzyme-linked immunosorbent assays (Amersham Pharmaceuticals) according to the manufacturer's instructions. The sensitivities of MMP-2, MMP-9, and TIMP-1 were 0.37 ng/mL, 0.6 ng/mL, and 1.25 ng/mL, respectively. Intra-assay variations were <10% for all the above assays.

Serum concentrations of interleukin (IL)-6, IL-8, tumor necrosis factor alpha (TNF*α*), and monocyte chemoattractant protein-1 (MCP-1) were analyzed using an ultrasensitive electrochemiluminescence immunoassay (Meso Scale Discovery). This assay was performed on a custom made 4-plex following instructions from the manufacturer. Plates were analyzed using a Meso Scale Discovery Sector Imager 2400 instrument. Duplicate measurements were performed in all the above tests.

### 2.3. Doppler-Echocardiography Study

Two-dimensional Doppler-echocardiography study was performed in the standard fashion. All data represent the mean of 3 measurements on sequential cardiac cycles. LVEF was calculated by the Teichholz method. *E/e*′ was calculated using tissue Doppler imaging and mitral valve Doppler. LAVI was calculated using biplane area length method as recommended by the American Society of Echocardiography Guidelines [20]. Systolic LV meridional wall stress was calculated accordingly: {0.334*Pd*}/{*h*[1 + (*h*/*d*)]}10^3^ dyn/cm^2^, where *P* is systolic cuff pressure (mmHg), *d* is systolic LV diameter (cm), and *h* is systolic posterior wall thickness (cm) [21]. All measurements were made by blinded observers.

### 2.4. Statistical Analysis

Data are presented as mean ± standard deviation or frequency and percentages. Biomarkers that were not normally distributed are presented as medians with interquartile ranges. BNP was dichotomised by the median cutoff value of 165 pg/mL. Group comparisons were made using two-sample independent *t*-test, chi-squared test, and Mann-Whitney *U* test where appropriate.

Multivariable analysis was performed via multiple linear regression of Log(BNP) with those independent variables that appeared significant in the univariate and correlation analysis. After initial parameter exploration, variable selection was focused on the emergent factors of age, indicators from echocardiography of ventricular function (LVEF) and fluid overload (*E/e*′), and one indicator from each of renal function (creatinine), inflammation (IL8), and collagen turnover (PIIINP). Robustness of model to the choice of collagen and inflammatory marker was further assessed by cycling through all combinations.

Magnitude of association of each covariate with BNP was assessed as follows. In a multiple linear regression model through Log(BNP) with independent parameter *x*_1_ having coefficient *b*_1_, an *n*-unit increase in *x*_1_ will be associated with an exp⁡(*n*∗*b*_1_) factor change in BNP independent of all other parameters in the model, using the law of logarithms that Log(*A*) − Log(*B*) ≡ Log(*A*/*B*).

Subsequent to these analyses, and based on the original observation of the independent relationship between markers of collagen turnover and BNP, a more detailed study was performed on the impact of other independent variables on this BNP-collagen relationship. For example, the relationship between BNP and the collagen markers was evaluated with reference to LVEF. To illustrate this, the population was dichotomised by median PIIINP and then median BNP in each dichotomy was observed in overlapping subsets of LVEF in the range of ±4(%) in either side of a specified integer value of LVEF. The progress of the median BNP was then plotted against the integer value as it was scanned across the EF range. This projection was similarly repeated for the other principal associates of BNP, namely, age ±4 (years), *E/e'* ± 3, and creatinine ±15 (mmol/L). Two-sided probability values reported with *P* < 0.05  are considered as statistically significant. All presented logarithms are natural logarithms. Statistical analysis and graphical modelling were performed on  *R*.

## 3. Results

### 3.1. Population Demographics and Univariate Associates of BNP

The demographic characteristics and clinical measurements of 179 stable HF patients according to median BNP cutoff value of 165 pg/mL are shown in Tables 1 and 2.

The mean age of the population was 71 ± 12 years and 63% were males. Patients with a BNP of >165 pg/mL were older and more likely to have ischemic etiology (68%). In the total population, 76% of patients had left ventricular systolic dysfunction (LVSD) (LVEF < 50%) and 24% had preserved systolic function HF (HF-PSF) at the time of entry into our HF program. Patients with a BNP of >165 pg/mL were more likely to have lower ejection fraction. 90% of those with LVSD were on dual disease modifying therapy including either angiotensin converting enzyme inhibitor or angiotensin receptor blocker combined with a beta blocker.

Age, BMI, left ventricular dimension in systole (LVIDs), LVEF, urea, creatinine, LAVI, and *E/e*′ were significant univariate associates of elevated BNP. Of the markers of collagen turnover, the extracellular matrix modifying enzymes MMP2 and TIMP1 were significantly associated at the *P* < 0.05 level and the collagen markers (PINP, PIIINP, and CITP) were significantly associated with elevated BNP except for PICP. The markers of inflammation were not significantly associated with elevated BNP.

### 3.2. Multivariable Analysis of BNP Association

Across all model instances, age, creatinine, LVEF, and *E/e*′ are strong, independent, multivariable associates of BNP, as are the markers of collagen turnover, except for PICP. This result is robust to choice of collagen marker and the change in *R* ^2^ between the models is relatively indifferent. The multiple linear regression model with Log(BNP) as the dependent variable and independent variables as age, creatinine (renal function), LVEF (ventricular function), *E/e* ^'^ (fluid overload), IL8 (inflammatory), and PINP/PIIINP/CITP/PICP (collagen) is given in Table 3.

To illustrate magnitude of association, consider the multiple linear model generated with PIIINP and IL8: Log(BNP) = 0.032 ∗ Age − 0.017 ∗ LVEF + 0.0044 ∗ Creatinine − 0.0035 ∗ IL8 + 0.093 ∗ PIIINP + 0.046 ∗ *E/e*′ + 2.12. In this model, clinically relevant changes in LVEF by −5%, *E/e*′ by +2.0, and PIIINP by +1.0 (*μ*g/L) are independently associated with factor increases in BNP of 1.089, 1.096, and 1.097, respectively. For comparison, a 5-year increase in age corresponds to a factor of 1.17. It is noted that replacing IL8 with other inflammatory markers failed to show significance of inflammatory variables in the presence of the aforementioned covariates (results not presented).

### 3.3. Deconstructing BNP and Markers of Collagen Turnover

**Figure 1:**
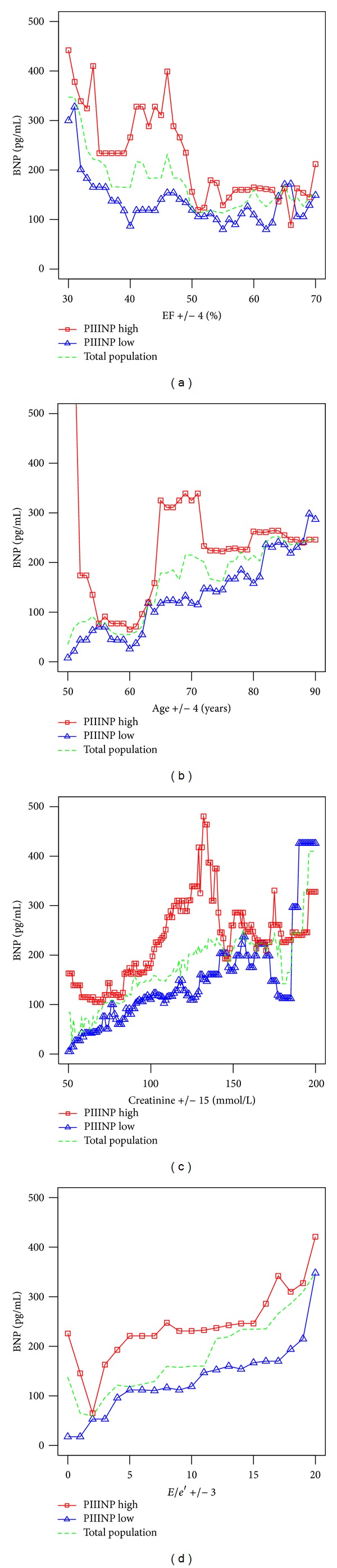
Projection of BNP onto its principal associates when population is dichotomized by median PIIINP of 4.72 (*μ*g/L).

**Figure 2:**
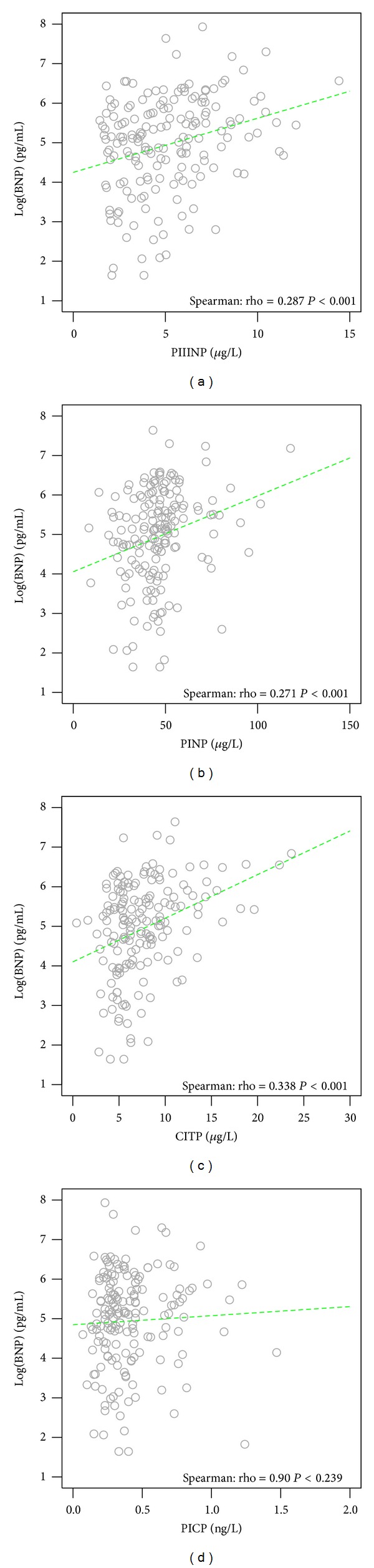
Correlation of collagen markers with Log(BNP).

**Figure 3:**
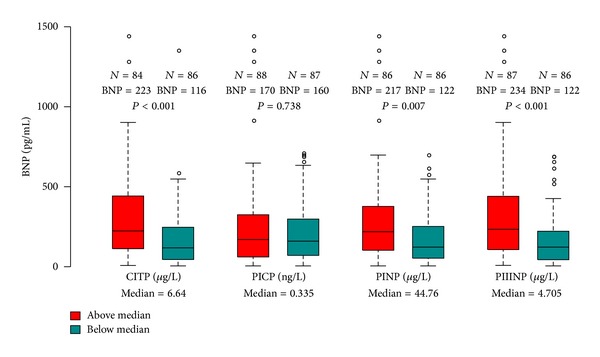
Boxplots of BNP (pg/mL) distribution when population is dichotomized by median values of collagen markers. Depicted labels indicate group size, median BNP, and Mann-Whitney *U* test statistical comparison.

Plots of median BNP deconstructed in the framework of LVEF, *E/e*′, age, and creatinine are given in Figure 1 for the total population and for the population split by the median PIIINP cutoff value of 4.72 (*μ*g/L). This demonstrates that, independent of LVEF, renal function, and *E/e*′, heightened markers of collagen turnover additionally contribute to increases in localised BNP expression. In isolation, the association of BNP with these markers is illustrated by the correlations of Figure 2 and by the boxplots of Figure 3.

## 4. Discussion

This study investigated the associates of elevated BNP in a stable heart failure population. As anticipated, the data demonstrate that age, renal function, and left ventricular ejection fraction are strong independent associates of BNP. Aside from the impact of these variables, the results also underline the importance of filling pressure, as assessed noninvasively by measurement of *E/e*′ and markers of collagen turnover. These results confirm the view that multiple stimuli may be relevant in driving BNP levels. Defining the relative importance of such stimuli in individual patients may guide more focused therapy of persistently elevated NP in clinically stable patients with possible further improvements in outcomes for natriuretic peptide (NP) guided therapy. Several datasets have convincingly demonstrated the prognostic value of NP levels in both the inpatient and outpatient management of HF [5]. In the hospital setting, the immediate level of NP on presentation to the emergency room with acute decompensated HF is an independent predictor of outcome [22], as is the early reduction in NP levels with treatment [23]. Furthermore, the level of discharge from hospital is a strong predictor of immediate recurrent events [24, 25]. NP levels in stable HF patients in the community are among the most accurate determinants of outcome [2–4], as are percent changes in levels over time [5, 26].

Given the power of NP in predicting outcome in these datasets, it is not surprising that the concept of using NP levels in addition to clinical features would be tested as a means of guiding therapeutic decisions in this population. Troughton and colleagues were the first to test this hypothesis, where in a single centre, randomised controlled study they demonstrated that NP-guided therapy resulted in a significant reduction in cardiovascular events and a delay in time to onset of a first cardiovascular event [7]. In the first multicentre study, Jourdain and colleagues demonstrated that BNP-guided therapy in an optimally treated population resulted in a significant reduction in HF-related death and hospitalisation compared to those managed by clinical features alone [6]. Subsequently, the TIME-CHF investigators addressed whether treatment guided by NT-proBNP compared with management directed by clinical features would be of benefit [8]. This group also addressed in a prespecified subanalysis whether the impact of NP-guided therapy would be different between those aged above and below the age of 75 years on entry into the study. Their observations demonstrated no overall benefit from this approach, but there was benefit in those less than 75 years with NP-guided therapy in terms of reduction of HF-related events. More recently, the BATTLESCARRED study was published on the relative impact of hormone-guided therapy versus intensive medical care and routine care. Improved longer-term mortality was observed in patients in the NT-proBNP-guided group compared with the two other groups, but again only in those aged <75 years on enrolment [9]. Finally, Berger et al. recently demonstrated that a NP-guided strategy reduced events following discharge compared with routine multidisciplinary care or routine care [27]. While these trials in the main present positive results and indicate that hormone-guided therapy with NP may be the first step towards individualisation of therapy in stable HF, some other observations from within these studies deserve mention. No information is provided regarding how individual therapies were chosen to reduce NP and it appears that there was a generic strategy in all of the above-quoted studies directed to maximise disease modifying therapies and diuretics. In other words, persistently elevated NP levels may simply act as a prompt to increase doses of drugs already prescribed rather than specifically to redirect therapy. Also, it is of interest that NP failed to achieve target levels in the majority of patients [6, 9]. These observations taken together suggest that a more complete understanding of the mechanisms underlying an elevated NP level may result in even more effective results from this strategy.

In patients with stable HF, there are many potential causes of an elevated NP level, including wall stress, myocardial fibrosis, inflammation, and ischemia [10–13]. In addition, even when clinical assessment indicates euvolaemia, occult fluid overload may be present, explaining the elevated NP levels [15]. With such varied reasons, it seems logical that understanding the specific or dominant stimulus for an elevated BNP may be important in order to choose the most effective therapy. The data presented here provide original information on the relative importance of a number of known stimuli for BNP in patients with clinically stable HF. As anticipated, left ventricular systolic function, age, and renal function were important determinants of NP. Of interest, however, was the independent role of surrogate measures of left ventricular filling pressure and myocardial interstitial disease. All patients in this study were deemed euvolaemic by a trained observer. Nonetheless, *E/e*′ was above 10 in 42% and above 15 in 9% despite the reassuring clinical exam. These observations would suggest that an objective measure of filling pressure should be obtained when assessing stable patients with persistently elevated BNP as it may indicate the need for an increase in diuretic therapy. An empiric increase in diuretic without such evidence may worsen renal function and thereby even increase BNP levels.

In addition to this, and independently of all other factors including *E/e*′ levels, patients with elevated BNP were found to have increased markers of collagen turnover with the exception of PICP. The clinical implication of this observation is that stable HF patients with elevated BNP levels may have more established abnormalities of the cardiac interstitium, likely characterised by increased collagen turnover leading to progressive ventricular dysfunction. Abnormalities of the cardiac interstitium have been shown to stimulate a NP response which may be a protective mechanism in attempt to modulate myocardial fibrosis as highlighted in experimental BNP and NPRA receptor knockout animal models [28, 29]. This link between heightened BNP levels and abnormalities of the cardiac interstitium has also been demonstrated in patients with diastolic heart failure [30]. It is well established that increased fibrosis may predispose to arrhythmia and also impair systolic and diastolic function of the LV, potentially contributing to the poor prognosis associated with an elevated BNP. Furthermore, this observation may indicate that these clinically stable patients with an elevated BNP would benefit from therapies which may more directly influence abnormalities of collagen turnover affecting the cardiac interstitium, such as aldosterone antagonists [31]. In this regard, it is of interest that the limited data available on the benefit of aldosterone antagonists in systolic dysfunction HF indicates that prognosis is improved only in individuals with elevated levels of markers of collagen turnover at baseline [32]. Furthermore, the only therapy not used with more frequency in the NP-directed arm in BATTLESCARRED was the aldosterone antagonist, possibly compromising the efficacy of this strategy in the study [9]. It is also noteworthy that emerging data suggest that PIIINP is a strong, independent prognosticator in HF [30–34]. It is possible therefore that in clinically stable heart failure patients with persistent elevation of BNP assessment of collagen turnover should be performed and where abnormal be used as an indicator for aldosterone antagonist therapy. Such a hypothesis requires examination, but if proven may be a first step towards individualisation of therapy for this patient population. Of note, in addition to NP-guided therapy, other disease-associated biomarkers are being proposed to help guide treatment strategies, and it is possible that a more elaborate biomarker panel may be of greater therapeutic benefit [35].

Based on the results from TIME and BATTLESCARRED, there is interest in the observation that those above 75 years of age did not seem to respond to NP-driven therapy. Our analysis of those above the median age of 73 years in this population indicates that despite the univariable associates remaining largely unchanged, when dichotomizing BNP by the group median of 219 pg/mL, the multivariable model fails, typically accounting for only 8% of the variation in BNP. For the younger cohort, significance remains as before. This may support the hypothesis that treatment responses to high BNP in older cohorts should differ from younger cohorts.

## 5. Limitations

In interpreting these data, it is important to note that this may be the first attempt to define Doppler echocardiography and biomarkers of collagen turnover and inflammation as associates of elevated NP levels in patients with stable HF, and therefore these results require confirmation. Furthermore, while this is a well-characterized population, the number of patients is relatively small, limiting our ability to more completely assess associates of elevated NP beyond what was analyzed. Also, the role of ischemia was not addressed beyond the presence of a predominant ischemic etiology which did not independently predict an elevated BNP. Of note, given the importance of NP-guided care in stage A/B HF, it would be of value to identify drivers of this NP signal in early disease prior to onset of symptomatic HF, which may in turn provide a more target-based therapeutic strategy in HF prevention [36]. Finally, the association of elevated BNP levels with heightened collagen formation has been dependent on analysis of serum levels of relevant markers. While accepted as an adequate surrogate of myocardial collagen content, and accepting that direct histological analysis may not be ethically possible, it would be of interest to image the myocardium with cardiac magnetic resonance imaging and quantify collagen content to get direct confirmation of this observation.

## 6. Conclusion

Persistent elevation of NP in clinically stable patients portends a poor outlook. Multiple mechanisms are likely responsible for increased levels of this biomarker. Defining these stimuli may be critical to effectively reduce the level of this peptide, defining a better prognosis. These data presented here provide original data on the relative importance of various factors and underline the potential role of persistent occult fluid overload and abnormalities of the cardiac interstitium. These observations may further refine NP-directed therapeutic strategies with potential for further improvements in outcome.

## Figures and Tables

**Table 1:** Table of demographics and clinical parameters for total population and for the population split by median BNP of 165 pg/mL.

	All	BNP > 165	BNP ≤ 165	*P*
Male	113 (63%)	56 (62%)	57 (64%)	0.92
Age (years)	71 ± 12	75 ± 11	67 ± 13	<0.001
BMI (kg/m^2^)	30 ± 7	28 ± 6	32 ± 8	0.009
Systolic BP (mmHg)	131 ± 19	131 ± 20	131 ± 19	0.90
Diastolic BP (mmHg)	71 ± 11	70 ± 12	72 ± 11	0.21
Idiopathic	30 (19%)	6 (8%)	24 (28%)	0.003
Ischaemic	86 (54%)	50 (68%)	36 (42%)	0.003
Hypertension	51 (32%)	29 (39%)	22 (26%)	0.10
Valvular	18 (11%)	10 (13%)	8 (9%)	0.57
Biochemistry				
BNP (pg/mL)	165 (71 : 325)	325 (231 : 516)	67 (27 : 115)	<0.001
Urea (mmol/L)	7.8 (6.1 : 11.4)	9.3 (7.3 : 12.8)	6.7 (5.3 : 8.9)	<0.001
Creatinine (mmol/L)	115 (94 : 145)	126 (103 : 157)	107 (87 : 133)	<0.001
Sodium (mmol/L)	138 ± 3	138 ± 3	138 ± 3	0.36
Potassium (mmol/L)	4.4 ± 0.4	4.4 ± 0.3	4.4 ± 0.4	0.54
Medications				
Beta blocker	143 (90%)	68 (92%)	75 (88%)	0.61
ACEI/ARB	151 (95%)	70 (95%)	81 (95%)	0.87
Diuretics	131 (82%)	65 (88%)	66 (78%)	0.14
Aldosterone antagonist	27 (17%)	12 (16%)	15 (18%)	0.99

Values are mean ± standard deviation or median (interquartile range); BNP: B-type natriuretic peptide; ACEI: angiotensin converting enzyme inhibitor; ARB: angiotensin receptor blocker; BMI: body mass index; SBP/DBP: systolic and diastolic blood pressure.

**Table 2:** Echocardiogram and collagen/inflammatory markers for total population and for population split by median BNP of 165 pg/mL.

	All	BNP > 165	BNP ≤ 165	*P*
Echocardiogram				
EF (%)	50 ± 14	47 ± 15	53 ± 13	0.004
E/e′	9.7 ± 4.4	10.8 ± 5.0	8.6 ± 3.5	0.003
LAVI	50 ± 18	56 ± 18	46 ± 17	0.007
LV wall stress	81 (58 : 116)	88 (64 : 122)	77 (59 : 103)	0.27
LVDD phase 1+	64 (69%)	31 (66%)	33 (72%)	0.70
Collagen markers				
CITP (ug/L)	6.62 (5.12 : 9.24)	7.73 (5.42 : 10.53)	6.2 (4.82 : 8.13)	0.005
PICP (ng/L)	0.34 (0.25 : 0.46)	0.36 (0.27 : 0.5)	0.33 (0.23 : 0.44)	0.10
PINP (ug/L)	45.02 (37.25 : 51.2)	48 (41.65 : 55.1)	43.27 (32.86 : 47.83)	<0.001
PIIINP (ug/L)	4.72 (3.09 : 6.6)	5.35 (3.3 : 7.17)	4.09 (2.92 : 5.93)	0.014
Collagen turnover markers				
TIMP-1 (*μ*g/L )	0.28 (0.21 : 0.44)	0.32 (0.23 : 0.45)	0.25 (0.19 : 0.4)	0.010
MMP-9 (*μ*g/L)	0.37 (0.22 : 0.58)	0.34 (0.2 : 0.54)	0.41 (0.24 : 0.6)	0.29
MMP-2 (*μ*g/L)	2.33 (1.96 : 2.91)	2.55 (2.11 : 3)	2.15 (1.83 : 2.74)	0.004
Inflammatory markers				
IL-6 (ng/mL)	9.61 (6.46 : 15.78)	10.27 (6.36 : 14.92)	9.12 (6.54 : 16.11)	0.76
IL-8 (ng/mL)	17.58 (12.08 : 27.27)	17.31 (11.89 : 26.45)	17.74 (12.17 : 28.45)	0.97
MCP-1 (ng/mL)	699 (429 : 961)	674 (422 : 922)	738 (460 : 1076)	0.16
TNFa (ng/mL)	3.89 (2.6 : 5.86)	3.96 (2.69 : 6.19)	3.87 (2.51 : 5.77)	0.53

Values are mean ± standard deviation or median (interquartile range); LAVI: left atrial volume index; LVDD: left ventricular diastolic dysfunction; EF: ejection fraction.

**Table 3:** Multiple linear regression models of Log(BNP) for choice of inflammatory marker as IL8 and all combinations of collagen markers.

Parameter	PINP as collagen marker		PIIINP as collagen marker		CITP as collagen marker		PICP as Collagen Marker	
	Estimate (95% CI)	*P*	Estimate (95% CI)	*P*	Estimate (95% CI)	*P*	Estimate (95% CI)	*P*
Intercept	1.73 (0.54 : 2.93)	0.005	2.12 (0.99 : 3.24)	<0.001	2.31 (1.18 : 3.45)	<0.001	2.50 (1.36 : 3.65)	<0.001
Age	0.032 (0.017 : 0.046)	<0.001	0.032 (0.017 : 0.046)	<0.001	0.032 (0.017 : 0.046)	<0.001	0.033 (0.018 : 0.048)	<0.001
LVEF	−0.018 (−0.030 : −0.006)	0.003	−0.017 (−0.028 : −0.004)	0.006	−0.018 (−0.030 : −0.006)	0.003	−0.019 (−0.031 : −0.008)	0.001
Creatinine	0.0054 (0.0012 : 0.0095)	0.012	0.0044 (0.0002 : 0.0087)	0.042	0.0034 (−0.0012 : 0.0081)	0.146	0.0049 (0.0005 : 0.0093)	0.030
IL8	−0.0011 (0.0082 : 0.006)	0.759	−0.0035 (−0.010 : 0.003)	0.335	−0.002 (−0.009 : 0.004)	0.516	−0.0025 (−0.0097 : 0.0047)	0.495
Collagen	0.016 (0.005 : 0.026)	0.003	0.093 (0.029 : 0.157)	0.005	0.056 (0.007 : 0.106)	0.026	−0.0023 (−0.715 : 0.711)	0.995
*E/e*′	0.047 (0.009 : 0.085)	0.016	0.046 (0.007 : 0.084)	0.019	0.048 (0.010 : 0.087)	0.015	0.051 (0.012 : 0.090)	0.010
Adjusted *R* ^2^	0.34		0.34		0.32		0.30	

LVEF: left ventricular ejection fraction; PINP: aminoterminal propeptide of procollagen type I; PIIINP: amino-terminal propeptide of procollagen type III; CITP: carboxy-terminal telopeptide of collagen type I; PICP: carboxyterminal propeptide of procollagen type I.
